# Sirolimus Release from Biodegradable Polymers for Coronary Stent Application: A Review

**DOI:** 10.3390/pharmaceutics14030492

**Published:** 2022-02-24

**Authors:** Wei Xu, Makoto Sasaki, Takuro Niidome

**Affiliations:** 1 Faculty of Advanced Science and Technology, Kumamoto University, Kumamoto 860-8555, Japan; m.sasaki@charlielab.co.jp (M.S.); niidome@kumamoto-u.ac.jp (T.N.); 2 Charlie Lab Inc., Kumamoto 860-8555, Japan

**Keywords:** sirolimus, biodegradable polymer, drug release, drug-eluting stent

## Abstract

Drug-eluting stents (DESs) are commonly used for the treatment of coronary artery disease. The evolution of the drug-eluting layer on the surface of the metal stent plays an important role in DES functionality. Here, the use of biodegradable polymers has emerged as an attractive strategy because it minimizes the occurrence of late thrombosis after stent implantation. Furthermore, understanding the drug-release behavior of DESs is also important for improving the safety and efficacy of stent treatments. Drug release from biodegradable polymers has attracted extensive research attention because biodegradable polymers with different properties show different drug-release behaviors. Molecular weight, composition, glass transition temperature, crystallinity, and the degradation rate are important properties affecting the behavior of polymers. Sirolimus is a conventional anti-proliferation drug and is the most widely used drug in DESs. Sirolimus-release behavior affects endothelialization and thrombosis formation after DES implantation. In this review, we focus on sirolimus release from biodegradable polymers, including synthetic and natural polymers widely used in the medical field. We hope this review will provide valuable up-to-date information on this subject and contribute to the further development of safe and efficient DESs.

## 1. Introduction

Percutaneous coronary interaction (PCI) is a minimally invasive non-surgical approach for treating vascular disease. Balloon-expanded bare-material stents (BMSs) are an innovative PCI technology that was developed in the 1980s [1]. BMSs are usually fabricated with metals such as stainless steel, cobalt-chromium, and platinum-chromium. These alloys exhibit properties suitable for supporting the stent shape and durability. However, the placement of BMSs causes endothelial injury and leads to in-stent restenosis [2,3,4]. Accordingly, drug-eluting stents (DESs) were developed as a means to reduce the rate of restenosis [5].

**Figure 1:**
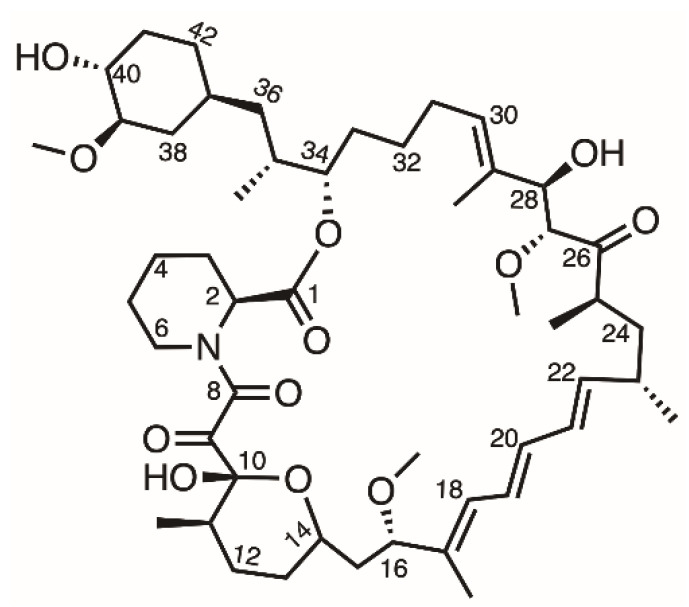
Structure of sirolimus.

The first-generation DES (Cypher, Cordis Corporation, Hialeah, FL, USA) was composed of a stainless-steel platform and a sirolimus-eluting durable-polymer layer [6]. Sirolimus is an antiproliferative drug that forms a complex with FK binding protein (FKBP-12) that then interacts with mammalian target of rapamycin (mTOR), which is involved in cell growth and proliferation (Figure 1) [7]. Due to the high permeability and poor water solubility (about 2.6 µg/mL) of sirolimus, it is classified as belonging to the BCS (biopharmaceutics classification system) class II drug category [8]. However, compared with other anti-proliferative drugs, sirolimus has better kinetics, a wider therapeutic index, and does not induce cell death even at higher concentrations [9]. Therefore, loading sirolimus onto a DES is a widely used method to inhibit the overgrowth of cells and reduce restenosis after stent implantation.

In the same year as the above system, a paclitaxel-eluting stent (Taxus, Boston Scientific, Marlborough, MA, USA) was approved as another first-generation DES. However, several studies showed that paclitaxel-eluting stents present a higher risk of in-stent lumen loss than sirolimus-eluting stents [10]. Therefore, sirolimus became recognized as the more suitable drug for stent treatment.

For long-term DES implantation (one or more years), newly occurring atherosclerotic processes called neoatherosclerosis and very-late in-stent restenosis were reported [11]. In addition to the health conditions of patients, the drug-release behavior and the biocompatibilities of the polymer and strut material of the platform emerged as significant risk factors [12,13]. Therefore, second-generation DESs were developed in 2008. Xience V (Abbott Vascular, Chicago, IL, USA) employed everolimus as the antiproliferative drug [14,15]. The platform material was changed to cobalt-chromium with a strut thickness of 81 µm (that of the Cypher stent was 140 µm), which was found to be more suitable for stent application. It was also reported that these thin-strut DESs resulted in 1.5 times less restenosis than thick-strut DESs [16]. Furthermore, in comparison with stainless steel, cobalt-chromium exhibits better flexibility, mechanical strength, and corrosion resistance.

Both the first- and second-generation DESs have durable polymers as their drug-eluting layers. However, it was reported that the durable polymer evoked a hypersensitivity reaction and thrombus formation after complete drug release [17]. To reduce the risk of thrombosis, patients were obliged to take antiplatelet drugs for a period of time [18]. Therefore, a new generation of DESs based on biodegradable polymers was developed to overcome the long-term risks involved with durable-polymer-coated DESs.

Biodegradable polymeric nanomaterials have been used for controlled drug delivery for many years. Such materials prolong the action of a loaded therapeutic agent and exhibit excellent biocompatibility [19]. Accordingly, a series of randomized trials demonstrated that biodegradable-polymer-coated DESs exhibit higher efficacy and safety than first-generation DESs and are non-inferior to second-generation DESs [20].

Regulating the drug-release behavior of DESs is a key factor to improving their performance. When applied correctly, it can inhibit excess cell growth, which is the main contributing factor to in-stent restenosis, without affecting normal endothelial functions [21]. However, biodegradable polymers with different properties present different drug-release behaviors. Molecular weight, composition, glass transition temperature, crystallinity, hydrophobicity, and the degradation rate are important properties affecting the behavior of polymers [22]. Additionally, the coating process employed, such as conformal coating or abluminal coating, and the solvent used also affect the drug-release behavior of biodegradable polymers.

After the development of second-generation DESs, studies on everolimus (a sirolimus analogue) were performed. However, several large randomized trials showed no significant differences in the rate of stent thrombosis and target lesion revascularization between the sirolimus-eluting stents and everolimus-eluting stents [23,24]. Therefore, the traditional antiproliferative-agent sirolimus is still generally used.

In this review, we summarize biodegradable polymers of poly-lactic acid, poly-D,L-lactic acid, and poly(lactic-*co*-glycolic acid), which have been used in the DES field over the past 20 years, and focus on the sirolimus release behavior not only from these synthetic polymers but also from natural polymers that are widely used in the medical field. In addition, we review several factors that affect sirolimus release from the polymers, such as the coating process, loading ratio of the drug, release medium, shear stress, pH, temperature, etc.

## 2. Synthetic Biodegradable Polymers

Biodegradable polymers are widely used in biomedical and pharmaceutical fields. They can be roughly divided into synthetic and natural biodegradable polymers [25]. Synthetic biodegradable polymers are commonly fabricated via condensation polymerization or ring-opening polymerization of monomers. Therefore, it is possible to control their molecular weights and physicochemical features by changing the monomer ratio and the fabrication process [26].

Here, we introduce several synthetic biodegradable polymers derived from lactic acid and glycolic acid that have been approved by the US Food and Drug Administration (FDA) for stent applications, and we discuss the sirolimus-release behavior of these polymers.

### 2.1. Poly-L-Lactic Acid

**Figure 2:**
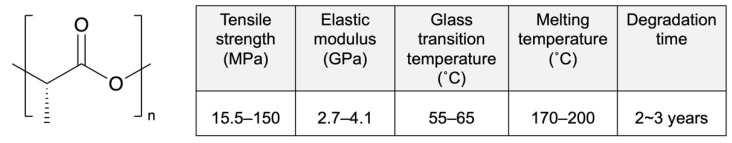
Structure and properties of poly-L-lactic acid (PLLA). Reprinted with permission from [31], published by Elsevier, 2016.

Poly-L-lactic acid (PLLA) is a biodegradable polymer that attracts ongoing research attention because of its excellent biocompatibility, high mechanical strength, and low cost (Figure 2) [27]. It is a semi-crystalline polymer with random and amorphous segments that is known for its high degree of crystallinity. The amorphous segments and molecular weight determine its degradation rate and influence its mechanical properties [28]. High-molecular-weight PLLA is used for clinal applications, especially stents, owing to its excellent mechanical properties [29]. Moreover, unlike low-molecular-weight PLLA (~80 kD), high-molecular-weight PLLA does not actively induce acute or chronic inflammation [30].

PLLA is employed as a drug-eluting layer or the platform of DESs approved by Conformité Européenne (CE) Mark and the FDA, as shown in Table 1. The molecular weight significantly affects the performance of DESs, but most information related to molecular weight is confidential. Excel (Jiwei Co. Ltd., Dongguan, China), composed of PLLA with sirolimus as the eluting layer, has shown superior outcomes in realtion to major adverse cardiac event compared with durable-polymer-coated DESs [32]. According to three-year clinical trials, the safety and efficacy of Excel in combination with six-month antiplatelet therapy has been demonstrated, and further evaluations are ongoing. Orisiro (Biotronik, Berlin, Germany) and BioMime (Meril Life Sciences, Gujarat, India) have PLLA and a PLLA co-polymer with poly-glycolic acid, respectively, for drug loading on metallic stents. After releasing the drug completely, the coating polymer degrades relatively quickly, whereas the metal platform remains in the vasculum. According to one-year clinical trials, Orisiro and BioMime present lower thrombosis rates than second-generation DESs [33]. Further trials are ongoing.

In our pervious study, we determined that high-crystallinity PLLA is an inferior drug-carrier because the lack of drug distribution in the crystalline phase leads to a burst drug release [34]. The burst release of the drug impairs the endothelization of DESs and leads to in-stent thrombosis. To decelerate the drug release from the polymer and improve the endothelization of DESs, several coating methods have been investigated. Illner et al. reported the fabrication of PLLA matrices with sirolimus via electrospinning [35]. PLLA was dissolved in a mixed solvent of chloroform and 2,2,2-trifluoroethanol (1/4, *v*/*v*). Compared with PLLA films, the use of PLLA fibers inhibited the burst release of sirolimus. It was demonstrated that the lower crystallinity of PLLA fibers led to better drug distribution in the PLLA structure. Thus, optimal drug release can be achieved by controlling the structure of the PLLA layer.

In recent years, PLLA has also been used as a platform for stents, as shown in Table 2. Here, we briefly mention bioabsorbable stents composed of PLLA, termed bioresorbable scaffolds (BRSs). The first-in-man application of a fully biodegradable stent was achieved with the Igaki-Tamai stent (Kyoto Medical Planning Co. Ltd., Kyoto, Japan), which is made of PLLA without drug loading [36]. Even though it showed good short-term results, low primary patency rates at 12 months were indicated by several non-randomized trials [37]. Therefore, the approach of loading the anti-proliferation drug on the stents is still general for stent treatment. The platforms for Absorb BVS (Abbott Vascular, Chicago, IL, USA), Xinsorb (Huaan Biotch., Laiwu, China) and MeRes 100 (Meril Life Sciences, Gujarat, India) are also made of PLLA and are coated with poly-D,L-lactic acid, containing siolimus as the drug-eluting layer [38,39]. However, stents made of PLLA have poorer mechanical properties than those made of metallic materials, and the thicker struts of PLLA DESs lead to a higher incidence of in-stent thrombosis [40]. As a result, the high incidence of in-stent thrombosis associated with Absorb BVS led to its withdrawal from the market [41].

### 2.2. Poly-D,L-Lactic Acid Used for Biodegradable DESs

**Figure 3:**
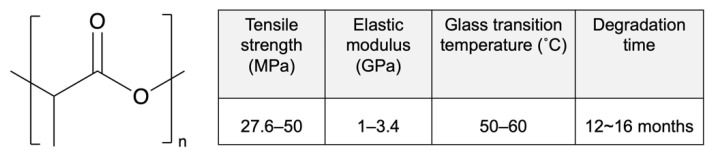
Structure and properties of poly-D,L-lactic acid (PDLLA). Reprinted with permission from [31], published by Elsevier, 2016.

Poly-D,L-lactic acid (PDLLA) is an amorphous polymer consisting of L-lactic acid and D-lactic acid monomers that is widely used in the DES field. PDLLA exhibits lower crystallinity and faster degradation than PLLA (Figure 3), and it is easier to fabricate, with less macroscopic phase separation because of its lower crystalline fraction [42]. In addition, the amorphous structure of PDLLA results in favorable distribution of the drug in the PDLLA structure for a prolonged release.

**Figure 4:**
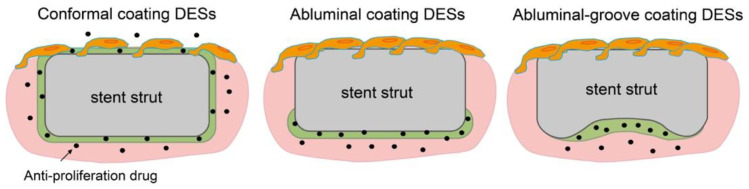
Schematics of endothelization on conformal coating, abluminal coating, and abluminal-groove coating DESs.

Table 3 shows several kinds of DESs fabricated with PDLLA that have been approved by CE Mark or are currently under evaluation (not limited to sirolimus). Regrettably, as above, the molecular weight information for PDLLA is confidential. Nobori (Terumo, Japan) has PDLLA and biolimus (a sirolimus analogue) coated on the outer surface by means of a novel method called abluminal coating [43]. Here, the drug-eluting layer is formed only on the side that is in contact with the blood vessel, as shown in Figure 4. This technique has been reported to be more suitable for endothelization than conformal coating [44]. Moreover, rapid endothelization also allows for shorter antiplatelet therapy, which results in a reduced risk of major bleeding. According to five-year clinical outcomes, Nobori presents lower risks of cardiac death and stent thrombosis than Orsiro, but further clinical results are pending [45]. Yukon Choice PC (Translumina Gmbh, Hechingen, Germany) is coated with PDLLA and shellac resin for sirolimus loading, and it has a microporous surface [46]. The microporous surface is expected to be better for endothelization. Compared with Cypher and Xience, Yukon Choice PC presents a lower incidence of stent thrombosis according to its five-year clinical outcomes. Firehawk (MicroPort Medical, Amsterdam, Netherlands) features abluminal-groove coating with PDLLA and sirolimus. The grooves on the outer surface of the stent prevent redundant drug loading and allow the targeted release of sirolimus, as shown in Figure 4 [47]. According to clinical outcomes, the safety and efficacy of Firehawk are non-inferior to those of Xience, but long-term clinical assessment remains necessary. Ultimaster (Terumo, Tokyo, Japan) is coated with the *co*-polymer poly(D,L-lactide-*co*-caprolactone and sirolimus using a gradient coating method [48]. The gradient coating decreases crack formation on the layer after expansion and reduces redundant drug loading. In our previous studies, we observed that expansion of PDLLA-coated stents causes critical defects, cracking, and desorption of the layer [40,49]. The gradient coating is expected to resolve this problem. Combo (OrbusNeich Medical, Hoevelaken, Netherlands) was designed to promote rapid endothelial formation with two therapeutic coating layers. An anti-restenosis abluminal layer and a pro-healing luminal layer contain sirolimus and anti-CD34^+^ antibodies, respectively, to promote endothelialization. Compared with Cypher, Combo shows better endothelial cell adhesion and lower rates of neointimal hyperplasia, but further evaluations are ongoing [50]. Thus, in this section, we demonstrated that an abluminal coating reduces redundant drug loading for better endothelization outcomes, commonly seen for new-generation DESs, and that they show lower rates of thrombosis and allow more effective treatment.

**Figure 5:**
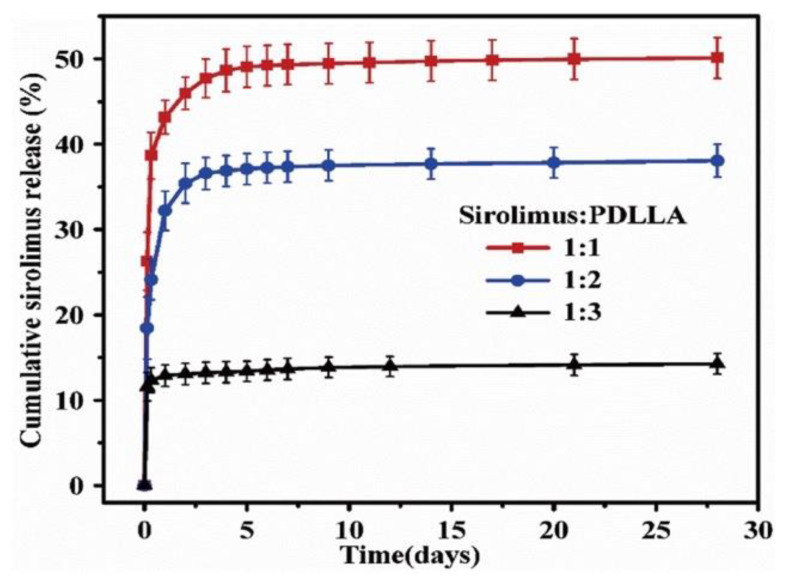
Cumulative sirolimus release profiles for poly-D,L-lactic acid (PDLLA) coatings with three different drug/polymer ratios in phosphate buffered saline solution at 37 °C. Adapted with permission from [51]; published by Elsevier, 2018.

**Figure 6:**
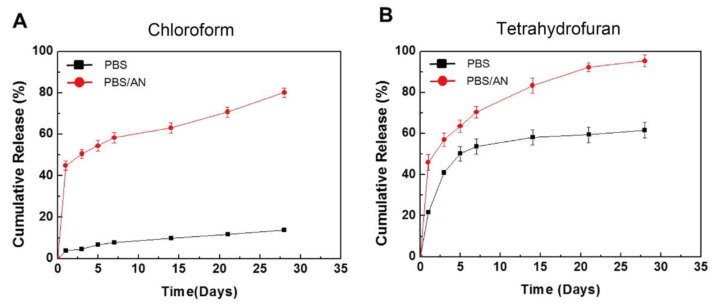
In vitro cumulative release of sirolimus from poly-D,L-lactic acid (PDLLA) layers in phosphate buffered saline (PBS) or PBS with acetonitrile (AN), prepared using an ultrasonic spray-coating system with (**A**) chloroform or (**B**) tetrahydrofuran. Adapted with permission from [52]; published by Elsevier, 2017.

To control the drug release from a polymer, not only the coating method, but also the drug/polymer ratio and organic solvent used are important. For instance, Li et al. reported the effects of various ratios of sirolimus and PDLLA on the release rate [51]. As shown in Figure 5, the sirolimus-release profiles exhibit two phases, i.e., a burst release for 1–3 d, followed by a slower sustained-release period for 28 d. Clearly, increasing the amount of sirolimus in the PDLLA accelerates the burst release. After seven days, all three coatings exhibit extremely slow release, which is due to the slow degradation rate of PDLLA and the diffusion-controlled release mechanism. In addition, Kim et al. studied layers of sirolimus and PDLLA prepared with different solvents, i.e., chloroform and tetrahydrofuran [52]. As shown in Figure 6, in PBS medium, the sirolimus-elution rate for the layer prepared with chloroform is slower than that prepared with tetrahydrofuran. Furthermore, the sirolimus release is accelerated by adding acetonitrile to the PBS medium. This is because sirolimus molecules become aggregated in chloroform, especially at higher concentrations. Therefore, the layer prepared with tetrahydrofuran exhibits better drug dispersion and thus a more controllable drug release.

### 2.3. Poly(lactic-co-glycolic acid)

**Figure 7:**
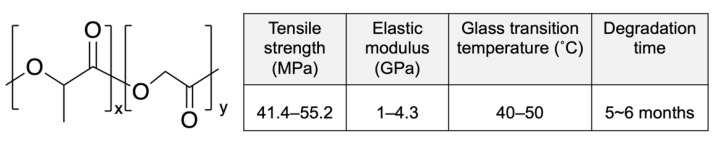
Structure and properties of poly(lactic-*co*-glycolic acid) (PLGA) (x-85L:y-15G). Adapted with permission from [31]; published by Elsevier, 2016.

Poly(lactic-*co*-glycolic acid) (PLGA), is a *co*-polymer composed of poly-lactic acid (PLA) and poly-glycolic acid (PGA). The physicochemical properties of PLGA can be controlled by changing the molar ratio of lactic acid and glycolic acid in the polymer chains [53]. When the crystalline glycolic acid is *co*-polymerized with lactic acid, the crystallinity of PGA is reduced. Therefore, a high content of glycolic acid in PLGA leads to fast degradation. However, as an exception, lactic acid/glycolic acid at a ratio of 50:50 exhibits the fastest degradation [54]. The structure and properties of PLGA (85L:15G) are shown in Figure 7. Compared with PLLA and PDLLA, PLGA has a lower glass transition temperature (Tg) and faster degradation.

Several kinds of DESs featuring a PLGA layer that have been approved by CE Mark or under ongoing evaluation are shown in Table 4 (not limited to sirolimus). Tivoli (Essen Tech., China) has a conformal coating with PLGA and sirolimus. The safety and efficacy of Tivoli for one year compared with durable-polymer-coated DESs has been confirmed by clinical trials [55]. However, compared with Xinsorb, incidences of late-target lesion failure and thrombosis are higher at 12 months [56]. Synergy (Boston Scientific, USA) consists of a thin-strut platinum-chromium stent platform with a PLGA and everolimus coating. The coating method used the abluminal-rollcoat method to reduce the total polymer burden and eliminate long-term exposure to late thrombosis [57]. According to large one-year real-life-population clinical trials, Synergy appears to be safe and effective, with low rates of restenosis (compared with those for Orisiro, Xience, and Ultimaster) owing to its thin-strut and abluminal coating. However, long-term studies are necessary [58]. Mistent (Micell Technologies, North Carolina, USA) is similar to Tivoli. It is coated with PLGA and crystalline sirolimus using a dry-powder electrostatic coating process. The unique characteristic of Mistent is that the PLGA coating layer degrades within 90 days and the sirolimus is released completely within 45 days. However, the sirolimus is present in the tissue for 270 days, even though the polymer has disappeared [59]. This is due to the unique coating process and the crystalline properties of sirolimus. According to three-year clinical trials, early safety and efficacy have been confirmed when compared with Xinence [60]. In addition, the stent thrombosis risk for Mistent is significantly lower than that for Tivoli at 12 months. BuMa (Sino Medical, Rotterdam, Netherlands) comprises a stent platform with a PLGA and sirolimus coating, and an electro-grafted layer of poly-butyl methacrylate (PBMA) is added between the polymer and the stent platform for resistance to flaking, peeling, and cracking [61]. According to two-year clinical trials, BuMa exhibits superior endothelization and presents a lower incidence of stent thrombosis compared with Excel [62]. Further clinical trials are ongoing. Compared with PLLA and PDLLA, the fast degradation of the PLGA coating is expected to reduce the incidence of very-late thrombosis. However, several studies have demonstrated that the fast degradation of PLGA induces arterial inflammation owing to its acidic products and consequent pH effects [63].

**Figure 8:**
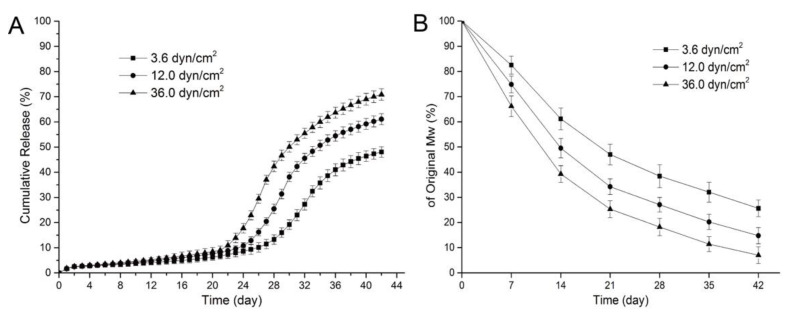
Release curves for sirolimus from poly(lactic-*co*-glycolic acid) (PLGA) for various shear stresses (**A**) and variations in the molecular weight of sirolimus-carrying PLGA films over time under various shear stresses (**B**). Adapted with permission from [66]; published by MDPI, 2017.

To reduce the inflammation induced by PLGA, the controlled-degradation and burst release of the drug from PLGA were studied. The degradation and drug release for PLGA depend on its physical properties, such as the monomer ratio, crystallinity, and molecular weight [64,65]. Moreover, the influence of environmental factors such as the degradation media, enzymes, and mechanical stress should also be considered. For instance, Zheng et al. investigated the effect of fluid shear stress on the degradation rate and sirolimus release from a PLGA film [66]. As shown in Figure 8A, all the samples showed a slow release of sirolimus for 19 d. After 20 d, an acceleration of the drug release was observed, and higher shear stress caused an earlier and faster sirolimus release from the PLGA film. This is because the higher shear stress leads to faster degradation of PLGA and affects drug diffusion and release (Figure 8B). Moreover, Abbasnezhad et al. investigated the effects of the medium flow rate on the drug release behavior of drug-loaded PLGA (LA/GA 50:50) film [67]. Increasing the flow rate of the medium leads to decreased mechanical stress for the PLGA film and significantly accelerates the burst drug release (~4-fold) from the PLGA film. Therefore, it is not only the intrinsic properties of drug-loaded polymers, but also their mechanical environment that influence the drug-release behavior of DESs [68,69].

## 3. Natural Biodegradable Polymers

Compared with synthetic biodegradable polymers, natural biodegradable polymers exhibit higher biocompatibility owing to their having similar macromolecular structures to natural molecules. However, they are sensitive to environmental factors such as temperature, pH, and mechanical stress [70]. Natural biodegradable polymers can be classified as proteins, polysaccharides, or polynucleotides [71]. Here, we focus on the proteins collagen and silk fibroin, which have been studied for biomedical applications because of their unique mechanical strengths, controllable degradation, and stability.

### 3.1. Collagen

**Figure 9:**
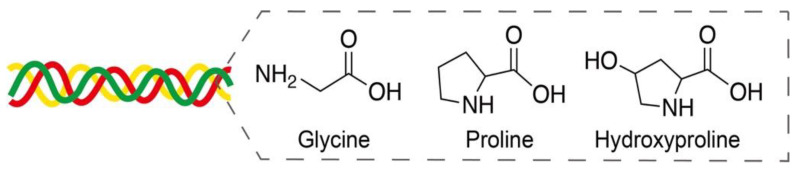
Amino acids glycine, proline, and hydroxyproline in a triplex helix collagen structure. Adapted with permission from [73]; published by MDPI, 2020.

Collagen, as the major component of extracellular matrices, provides mechanical support to connective tissues and is widely used in tissue engineering, wound healing, and bone/nerve regeneration applications [72]. Collagen is mainly composed of glycine, proline, and hydroxyproline in a triplex helix structure (Figure 9) [73]. Nearly 28 types of collagens have been identified, and type-I collagen is the most common in tissues. Owing to its excellent biocompatibility and degradation, collagen layers on the surface of metal stents are expected to provide improved thrombosis prevention and accelerated endothelialization after implantation [74].

**Figure 10:**
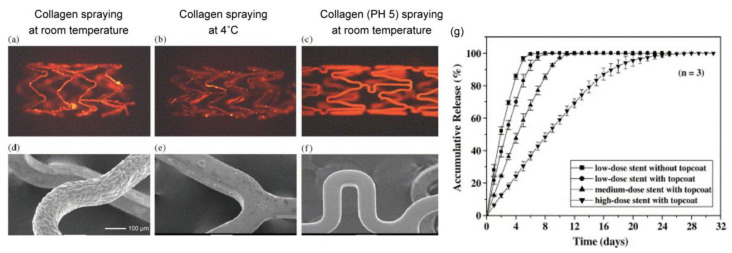
Fluorescence microscopic images and SEM micrographs of metallic stents spray-coated with collagen using different processes. (**a**,**d**) Neutral aqueous collagen spraying at room temperature; (**b**,**e**) neutral aqueous collagen spraying at 4 °C; and (**c**,**f**) aqueous collagen (pH 5.0) spraying at room temperature. (**g**) Cumulative release profiles for sirolimus from different types of the sirolimus-loaded stents. Adapted with permission from [75]; published by Elsevier, 2005.

Chen et al. first reported a stainless-steel stent coated with collagen (type-I) and sirolimus that was prepared with a spray method, and the optimal coating conditions were investigated [75]. As shown in Figure 10, collagen at pH 5.0 provided a uniform coating on the stents. Conversely, neutral collagen (pH 7.0) gradually gelled in the air brush and thus interfered with the spraying process, and collagen under low-temperature conditions reduced adhesion on the stents. To slow the sirolimus release from the collagen, an additional topcoat of collagen was applied. The release behavior was determined by the content of sirolimus in the collagen, with a higher dose of sirolimus in the collagen layer exhibiting a significantly slower drug release. Interestingly, this behavior is opposite to the sirolimus release from PDLLA (Figure 5). This is a noteworthy difference between synthetic and natural biodegradable polymers. Based on other studies on the drug release from collagen, we speculated that the interaction between the collagen and the sirolimus results in a slower drug release [76,77]. Although it is possible to control the drug release from collagen, several studies have demonstrated that collagen causes platelet adhesion, activation, and aggregation to induce further thrombosis formation, which is a concern in terms of late restenosis after complete drug release [78]. To address this problem, Yang et al. synthesized a recombinant human type-III collagen containing peptide triplets that provides potent cell adhesion activity and inhibits platelet adhesion [79]. After implanting the recombinant collagen-coated stents in the abdominal aortas of rabbits, the promotion of in-situ endothelialization and the inhibition of neointima hyperplasia were observed in a three-month evaluation in vivo. Since the anti-proliferation drug was not loaded in the collagen coating, it is expected that a highly biocompatible stent without an anti-proliferation drug could exhibit good performance.

### 3.2. Silk Fibroin

Among the natural polymers other than collagen used for tissue engineering applications, structural protein silk fibroin has shown great potential. The advantages of using silk for artificial blood vessels are its appropriate mechanical properties, predictable degradation products, and good biocompatibility [80]. Silk fibroin consists of heavy chains (∼390 kDa) and light chains (∼25 kDa) linked by disulfide bonds. The structure of the heavy chain consists of 12 hydrophobic domains, with 11 hydrophilic domains. The heavy chain forms β-sheet structures, which are mainly responsible for the excellent mechanical properties of silk fibroin [81].

**Figure 11:**
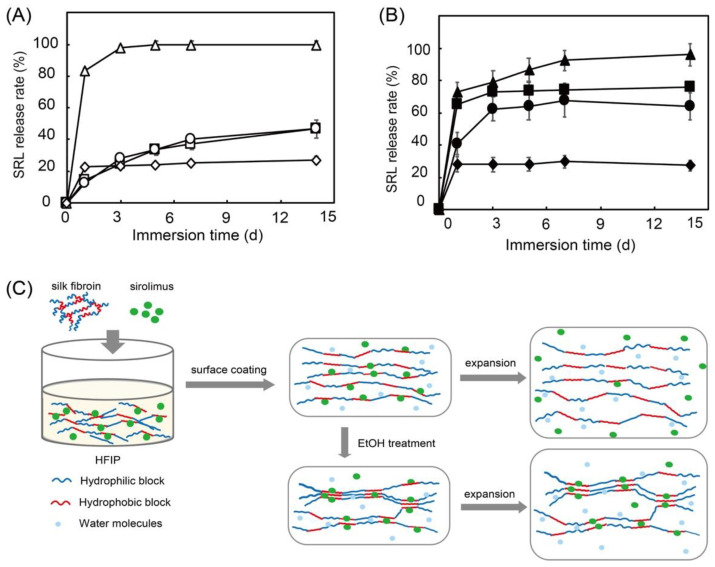
Sirolimus release over 14 days from silk fibroin (squares), silk fibroin with ethanol treatment (circles), poly-D,L-lactic acid (diamonds), and poly-caprolactone (triangles) on Co-Cr stents before (**A**) and after (**B**) balloon expansion in 37 °C phosphate buffered saline. Hypothesized mechanism of sirolimus release from silk-fibroin-coated stents before and after balloon expansion (**C**). Adapted with permission from [82]; published by American Chemical Society, 2020.

In a previous study, we used sirolimus-loaded silk fibroin as a surface coating for stents and evaluated the sirolimus release with/without balloon expansion [82]. Stents need to be expanded using a balloon catheter for placement in blood vessels. This process causes mechanical stress on the stent and affects DES performance. Moreover, ethanol treatment has been reported to influence the crystallinity of silk fibroin. Therefore, we investigated the sirolimus release with/without ethanol treatment [83]. As shown in Figure 11, without balloon expansion, silk fibroin exhibits a slow release regardless of ethanol treatment. However, with balloon expansion, a burst release of sirolimus is observed, and ethanol treatment of the silk fibroin suppresses the burst release at day 1. This is because ethanol treatment enriches the β-sheet structure and forms crystalline domains in the silk fibroin, which suppresses the burst release.

Interestingly, synthetic biodegradable PDLLA and poly-caprolactone show no differences with or without balloon expansion. The acceleration of the sirolimus release from silk fibroin with balloon expansion indicates that plastic deformation of the silk fibroin layer loosens the interaction between silk fibroin and sirolimus, as shown in Figure 11C. A similar previous study by Lee et al. also demonstrated that sirolimus is slowly released from silk fibroin microneedle wraps owing to the interaction between the sirolimus and the silk fibroin [84]. In addition, the adhesion of human umbilical vein endothelial cells and platelets to silk fibroin was evaluated. It shows excellent endothelial cell adhesion and minimal platelet adhesion. Compared with collagen, we confirmed that silk fibroin not only exhibits excellent drug-release behavior, it also shows high biocompatibility and decreased platelet adhesion. This is preferential for cardiovascular applications. However, mechanical stress and environmental conditions, such as the pH and temperature of the medium, which significantly affect the drug-release behavior of silk fibroin, should be considered in relation to its application to DESs [85].

## 4. Conclusions and Future Prospects

The effectiveness of DES therapy is largely dependent on the drug, coating polymer, and coating method because these factors significantly influence drug-release behavior and the risks of thrombosis and restenosis. Biodegradable polymers are expected to overcome the long-term risks associated with durable polymer-coated DESs. As synthetic biodegradable polymers, PLLA, PDLLA, and PLGA are widely used in DESs owing to their controllable mechanical and chemical properties. However, striking a suitable balance between a long-lasting drug release, fast endothelialization, and suitable degradation remains difficult to attain for biodegradable DESs. The use of natural proteins for DESs is gaining acceptance owing to their excellent biocompatibilities, faster endothelialization, and lower risk of thrombosis. However, environmental conditions such as medium pH and temperature significantly affect the properties of proteins and affect their drug-release behavior. Moreover, bioresorbable metals such as magnesium alloy and zinc alloy with superior mechanical properties are also expected to offer revolutionary alternative scaffolds to traditional DESs. Although optimal DESs capable of efficient treatment are still required, the new generation of DESs has significantly improved the safety and efficacy of stent treatments. In the future, DESs with superior performance are expected.

## Figures and Tables

**Table 1:** DESs with poly-L-lactic acid (PLLA).

Stent	Platform	Coating	Drug	Drug-Release Rate	Absorption Time of the Polymer (Months)	Molecular Weight of PLLA	Ref.
Excel	316L SS	PLLA	Sirolimus 14–15 µg/mm	180 d, 100%	6–9	confidential	[32]
BioMime	Co-Cr	PLLA + PLGA	Sirolimus 1.25 µg/mm^2^	30 d, 100%	2	confidential	[33]
Orsiro	Co-Cr	Silicon carbide + PLLA	Sirolimus 1.4 µg/mm^2^	30 d, 45%	14	confidential	

DES, drug eluting stent; Co-Cr, cobalt-chromium; PDLLA, poly-D,L-lactic acid; PLGA, poly(lactic-*co*-glycolic acid).

**Table 2:** Bioresorbable scaffolds (BRSs) with poly-L-lactic acid (PLLA).

Stent	Platform	Coating	Drug	Drug-Release Rate	Absorption Time of Polymer (Months)	Molecular Weight of PLLA	Ref.
Igaki-Tamai	PLLA	None	None	-	24	183 kDa	[36]
Xinsorb	PLLA	PLLA + PDLLA	Sirolimus 12 µg/mm	28 d, 83%	24–36	300 kDa	[37]
MeRes 100	PLLA	PDLLA	Sirolimus 1.25 µg/mm^2^	30 d, 82%	24	200–220 kDa	[39]
Absorb BVS	PLLA	PDLLA	Everolimus 100 µg/cm^2^	28 d, 80%	24	confidential	[41]

DES, drug-eluting stent; 316L SS, 316L stainless steel; Co-Cr, cobalt-chromium; PDLLA, poly-D,L-lactic acid; PLGA, poly(lactic-*co*-glycolic acid).

**Table 3:** DESs with poly-D,L-lactic acid (PDLLA).

Stent	Platform	Coating	Drug	Drug Release Rate	Absorption Time of Polymer (Months)	Ref.
Nobori	316L SS	Palylene + PDLLA abluminal	Biolimus A9 15.6 µg/mm	180 d, 100%	6–9	[43]
Yukon choice PC	316L SS	Shellac + PDLLA microporous	Sirolimus 4.8 µg/mm^2^	30 d, 90%	2–3	[46]
Firehawk	Co-Cr	PDLLA abluminal-groove	Sirolimus 0.3 µg/mm^2^	30 d, 75%	6–9	[47]
Ultimaster	Co-Cr	PDLLA-PCL gradient abluminal	Sirolimus 3.9 µg/mm	90~120 d, 100%	3–4	[48]
Combo	316L SS	PDLLA + PLGA with anti-CD34^+^ abluminal	Sirolimus 5 µg/mm	35 d, 100%	3	[50]

DES, drug-eluting stent; 316L SS, 316L stainless steel; Co-Cr, cobalt-chromium; PCL, poly-caprolactone; PLGA, poly(lactic-*co*-glycolic acid); anti-CD34^+^, antibody CD34^+^.

**Table 4:** DESs with poly(lactic-*co*-glycolic acid) (PLGA).

Stent	Platform	Coating	Drug	Drug Release Rate	Absorption Time of Polymer (Months)	Ref.
Tivoli	Co-Cr	PLGA	Sirolimus 8 µg/mm	30 d, 80%	3–6	[55]
Synergy	Pt-Cr	PLGA abluminal-rollcoat	Everolimus 100 µg/cm^2^	90–120 d, 100%	4	[57]
Mistent	Co-Cr	PLGA dry-powder electrostatic coating	Sirolimus 2.4 µg/mm^2^	45 d, 97% 270 d, 100% (in tissue)	3	[59]
BuMa	316L SS	PLGA electro-grafting	Sirolimus 1.4 µg/mm^2^	30 d, 100%	2	[61]

DES, drug-eluting stent; Co-Cr, cobalt-chromium; Pt-Cr, platinum-chromium; 316L SS, 316L stainless steel.

## Data Availability

Not applicable.
